# Tetrandrine Attenuated Doxorubicin-Induced Acute Cardiac Injury in Mice

**DOI:** 10.1155/2020/2616024

**Published:** 2020-05-08

**Authors:** Gang Li, Wen-Rui Li, Ya-Ge Jin, Qi-Qiang Jie, Cheng-Yu Wang, Lin Wu

**Affiliations:** ^1^Department of Cardiology, Peking University First Hospital, Beijing, China; ^2^Department of Ultrasound, The First Affiliated Hospital of Zhengzhou University, Zhengzhou, China; ^3^Department of Cardiology, The First Affiliated Hospital of Zhengzhou University, Zhengzhou, China; ^4^Institute of Cardiac Electrophysiology, Southwest Medical University, Luzhou, China

## Abstract

Oxidative damage is closely involved in the development of doxorubicin- (DOX-) induced cardiotoxicity. It has been reported that tetrandrine can prevent the development of cardiac hypertrophy by suppressing reactive oxygen species- (ROS-) dependent signaling pathways in mice. However, whether tetrandrine could attenuate DOX-related cardiotoxicity remains unclear. To explore the protective effect of tetrandrine, mice were orally given a dose of tetrandrine (50 mg/kg) for 4 days beginning one day before DOX injection. To induce acute cardiac injury, the mice were exposed to a single intraperitoneal injection of DOX (15 mg/kg). The data in our study showed that tetrandrine prevented DOX-related whole-body wasting and heart atrophy, decreased markers of cardiac injury, and improved cardiac function in mice. Moreover, tetrandrine supplementation protected the mice against oxidative damage and myocardial apoptotic death. Tetrandrine supplementation also reduced ROS production and improved cell viability after DOX exposure in vitro. We also found that tetrandrine supplementation increased nuclear factor (erythroid-derived 2)-like 2 (Nrf2) expression and activity in vivo and in vitro. The protection of tetrandrine supplementation was blocked by Nrf2 deficiency in mice. In conclusion, our study found that tetrandrine could improve cardiac function and prevent the development of DOX-related cardiac injury through activation of Nrf2.

## 1. Introduction

Doxorubicin (DOX) is a quinone-containing anthracycline and is widely used in the therapy of solid and hematologic malignancies. The clinical use of DOX triggers irreversible myocardial dysfunction, dilated cardiomyopathy, and heart failure [1]. Currently, dexrazoxane is the only agent that has been approved to reduce the toxic effects of DOX. However, the use of this drug may compromise the anticancer activity of DOX, which largely limits its clinical use [2, 3].

The precise mechanism of DOX-related cardiac injury is multifactorial, including increased reactive oxygen species (ROS) production, inflammatory response, and apoptotic cardiomyocyte death [4, 5]. It has been reported that the heart is more sensitive to DOX-related oxidative injury [6]. The production of ROS and subsequent lipid peroxidation were found in cardiac samples within three hours after DOX exposure [7]. Moreover, the suppression of ROS production by metallothionein rescued DOX-induced cardiotoxicity and improved cardiac function [8]. Thus, the search for a drug that could reduce oxidative stress in response to DOX is of great clinical importance for the treatment of DOX-related cardiac toxicity.

Tetrandrine is a bisbenzylisoquinoline alkaloid extracted from the root of *Stephania tetrandra* S. Moore. This drug has been clinically used for the treatment of hypertension in China [9]. Tetrandrine exhibited a broad range of pharmacological actions, including antitumor activity [10]. It has been reported that tetrandrine suppresses the tumor growth of human colorectal cancer through the inhibition of *β*-catenin [11]. A study reported that tetrandrine could reverse human cardiac myofibroblast activation and myocardial fibrosis [12]. In addition, tetrandrine inhibited the development of cardiac hypertrophy by suppressing the ROS-dependent signaling pathway in mice [13]. However, the effects of tetrandrine on DOX-induced cardiac injury, especially acute injury, and the related signaling mechanisms are unclear. Here, we first determined the protective effects of tetrandrine on DOX-induced acute cardiac injury in mice. We found that tetrandrine could suppress the acute toxic effects caused by DOX injection in mice.

## 2. Materials and Methods

### 2.1. Chemicals and Materials

The antibodies used to recognize nuclear factor (erythroid-derived 2)-like 2 (Nrf2, 1 : 1000, ab62352), GAPDH (1 : 1000, ab181602), proliferating cell nuclear antigen (PCNA, 1 : 1000, ab29), Bax (1 : 1000, ab32503), and cleaved caspase3 (1 : 1000, ab2302) were ordered from Abcam (Cambridge, UK). Tetrandrine (#Y0001165, >98% purity) and DOX were ordered from Sigma-Aldrich (Santa Clara, CA, USA). The bicinchoninic acid (BCA) protein assay kit was obtained from Thermo Scientific (Boston, USA). A cellular ROS assay kit (ab113851) was provided by Abcam (Cambridge, UK). Lactate dehydrogenase (LDH), total superoxide dismutase (SOD), glutathione (GSH), and glutathione peroxidase (Gpx) activity detection kits were purchased from Nanjing Jiancheng Institute of Biotechnology (Nanjing, China).

### 2.2. Experimental Animals

The animal experiment process was approved by the Institutional Animal Care and Use Committees at Peking University First Hospital (Beijing, China). The C57BL/6J male mice (8-10 weeks old, 21-25 g) used in our experiments were provided by Hunan STJ Laboratory Animal Co., Ltd. (Changsha, Hunan). They were housed in the Animal Care of Peking University First Hospital with free access to food and water. The mice were acclimatized for more than one week prior to experimental use. All animal treatments and subsequent analyses were performed in a blinded fashion. Tetrandrine suspensions were freshly prepared with 0.5% carboxy methylcellulose. To explore the protective effect of tetrandrine, the mice were orally given a dose of tetrandrine (50 mg/kg, 0.2 ml) for 4 days beginning one day before DOX injection. The control group was given the same volume of vehicle. The dose of tetrandrine for the animal experiment was selected according to a previous study [13]. To induce acute cardiac injury, the mice were exposed to a single intraperitoneal injection of DOX (15 mg/kg) or an equal volume of normal saline (NS) as a control [14]. All mice were weighed daily and sacrificed at 3 days post DOX insult. To deplete cardiac Nrf2, an adeno-associated virus 9 (AAV9) carrying shRNA targeting Nrf2 under the cTnT promoter (AAV9-shNrf2) or a negative control (AAV9-shRNA) was generated by HanBio Technology (Shanghai, China). The mice were subjected to a single intravenous injection of AAV9-shNrf2 or AAV9-shRNA via the tail vein at a concentration of 5 × 10^11^ viral genome per mouse [15, 16]. Six weeks after AAV9 injection, DOX was injected.

### 2.3. Hemodynamics

To evaluate cardiac function, hemodynamic analysis was carried out. The mice were anaesthetized with 1.5% isoflurane. Hemodynamic monitoring was performed using a Millar pressure-volume conductance system (Millar Instruments, TX) [17], which was inserted into the left ventricle. Hemodynamic parameters were obtained and analyzed by IOX software (emkaTECH).

### 2.4. Western Blotting

The total proteins from collected cardiac samples were extracted using RIPA lysis buffer. Equal amounts of protein were subjected to 10% SDS-PAGE. Then, the proteins were transferred to PVDF membranes and reacted with primary antibodies overnight at 4°C with primary antibodies. After incubation with the secondary antibodies, the blots were visualized using chemiluminescence (LumiGLO, Cell Signaling) and were quantified with a calibrated imaging densitometer (LAS-3000IR, Fujifilm).

### 2.5. RNA Extraction and Real-Time RT-PCR

Total mRNA was extracted from the mouse left ventricle using Ultra TurraxT8 (IKA Labortechinik, Staufen, Germany) with TRIzol reagent (Invitrogen Corp., CA). cDNA synthesis was performed by reverse transcription using the TransStart Top Green qPCR SuperMix kit. The expression of the indicated gene was amplified using SYBR Green PCR Master Mix (Applied Biosystems) and normalized to the expression of GAPDH.

### 2.6. Detection of Cardiac Injury Markers

Two days after DOX injection, blood samples were collected from the retro-orbital plexus of anesthetized mice. Blood was then centrifuged, and the supernatant was collected for further analysis. A mouse cardiac troponin-I ELISA kit (cTnI, Life Diagnostics) was used to measure the level of cTnI after DOX exposure. N-terminal probrain natriuretic peptide (NT-proBNP) levels were measured by an assay kit purchased from MyBioSource (CA, USA). We also used an LHD detection kit (Nanjing Jiancheng Institute of Biotechnology) to analyze cardiac injury.

### 2.7. Determination of Myocardial SOD, Gpx, 4-HNE, and GSH Content

Total SOD activity, MnSOD activity, and Gpx activity were evaluated to reflect the oxidative injury of the indicated groups using commercial kits. A reduced glutathione (GSH) assay kit and oxidized GSH assay kit were also used. The malondialdehyde (MDA) level was determined by a kit purchased from Nanjing Jiancheng Bioengineering Institute (Nanjing, China). The 4-HNE detection kit was purchased from Abcam. A caspase3 activity assay kit (based on spectrophotometry, Beyotime Biotechnology) was used to evaluate the activity of caspase3. The production of hydrogen peroxide was measured using a hydrogen peroxide assay kit (Abcam, ab102500).

### 2.8. Nrf2 and NF-*κ*B Activation Assay

Frozen hearts or cells were homogenized with RIPA lysis buffer. Nuclear proteins were extracted using NE-PER™ Nuclear and Cytoplasmic Extraction Reagents (Invitrogen). Nrf2 binding activity and nuclear factor kappa-B (NF-*κ*B) binding activity were analyzed using TransAM® Nrf2 Activation Assay Kits or TransAM® NF-*κ*B Activation Assay Kits (Active Motif).

### 2.9. TUNEL Staining

Heart paraffin sections were stained with a terminal deoxynucleotidyl transferase-mediated nick-end labeling (TUNEL) apoptosis detection kit to assess myocardial apoptosis according to the manufacturer's instructions. This kit was purchased from Roche Diagnostics (Indianapolis).

### 2.10. Cell Culture

Neonatal rat cardiomyocytes (NRCMs) were prepared as previously described [18]. These cells were cultured in DMEM (Gibco) supplemented with 10% fetal bovine serum (FBS). After 48 hours, tetrandrine alone or tetrandrine followed by DOX (5 *μ*mol/l) was added to the medium, and the cultures were incubated for the indicated time. Viability was determined using the CCK-8 assay kit according to the manufacturer's protocol. To knock down Nrf2, commercial rat Nrf2 siRNA and control siRNA were purchased from Santa Cruz Biotechnology (Santa Cruz, CA, USA). The cells were transfected with control siRNA or siNrf2 (50 nmol/l) for 4 hours using Lipo6000™ transfection reagent. Cells were harvested after 48 hours of transfection for further experiments.

To analyze ROS, we used a commercial cellular ROS assay kit (ab186027), which provided an ultrasensitive fluorometric one-step ROS assay that could be performed in a convenient 96-well microtiter plate format.

### 2.11. Statistical Analysis

The results are expressed as the mean ± standard deviation. A *t*-test was performed to assess significant differences between two groups. When comparing differences between two more groups, one-way ANOVA followed by Tukey's post hoc test was employed. A value of *P* < 0.05 was considered to be statistically significant.

## 3. Result

### 3.1. Tetrandrine Attenuated DOX-Related Cardiac Injury In Vivo

**Figure 1:**
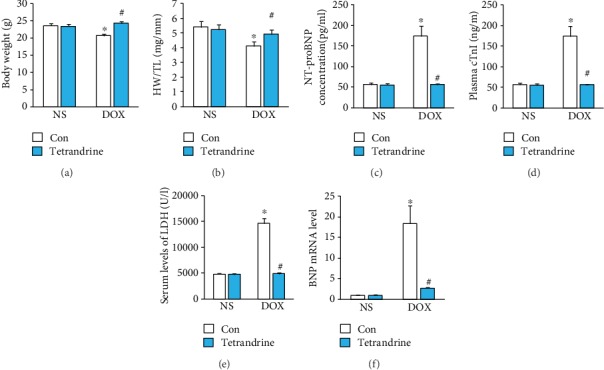
Tetrandrine treatment attenuated DOX-related cardiac injury in mice. (a) Body weight of animals in the indicated groups (*n* = 12). (b) The ratio of heart weight to tibial length (*n* = 12). (c–e) The levels of cTnI, NT-proBNP, and LDH in the indicated groups (*n* = 6). (f) The mRNA levels of BNP in mice (*n* = 6). ^∗^*P* < 0.05 compared with the NS group. ^#^*P* < 0.05 compared with mice after DOX injection.

In this experiment, mice were injected with a single dose of DOX to mimic DOX-related acute cardiac injury. As expected, mice treated with DOX alone demonstrated the classical decrease in body weight and heart weight to tibial length ratio compared with mice in the NS control group (Figures 1(a) and 1(b)). However, these pathological alterations were largely prevented by treatment with tetrandrine (Figures 1(a) and 1(b)). As shown in Figures 1(c) and 1(d), the levels of serum cTnI, NT-proBNP, and LDH were obviously increased in mice injected with DOX compared with those in the NS group, and these increases were suppressed by tetrandrine (Figures 1(c)–1(e)). Further analysis showed that the increased mRNA levels of brain natriuretic peptide (BNP) after DOX exposure were significantly reduced by tetrandrine treatment (Figure 1(f)).

### 3.2. Tetrandrine Improved Cardiac Function in Mice Injected with DOX

**Figure 2:**
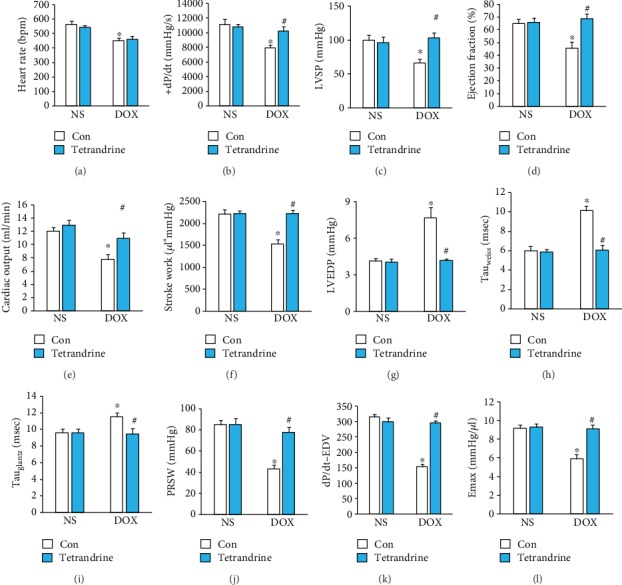
Tetrandrine treatment improved cardiac function in DOX-treated mice. (a) Heart rate in the indicated groups (*n* = 8). (b–d) Alterations in +*dP*/*dt*, LVSP, and EF in mice (*n* = 8). (e, f) Cardiac output and stroke work of mice (*n* = 8). (g–i) LVEDP and Tau values (*n* = 8). (j–l) PRSW, *dP*/*dt*-EDV, and Emax of the mice (*n* = 8). ^∗^*P* < 0.05 compared with the saline group. ^#^*P* < 0.05 compared with mice after DOX injection.

Tetrandrine treatment did not affect the heart rate in DOX-treated mice (Figure 2(a)). The administration of DOX resulted in a marked decrease in the maximum first derivative of ventricular pressure with respect to time (+*dP*/*dt*), left ventricular systolic pressure (LVSP), ejection fraction, stroke work, cardiac output, and load-independent indexes of contractility (preload recruitable stroke work (PRSW), *dP*/*dt*-end-diastolic volume relation (*dP*/*dt*-EDV), and end-systolic pressure-volume relation (Emax)) and an increase in the prolongation of relaxation time constants (Tau_Weiss_ and Tau_Glantz_). These changes were significantly attenuated by treatment with tetrandrine (Figures 2(b)–2(l)).

### 3.3. Tetrandrine Treatment Attenuated DOX-Induced Oxidative Stress in Mice

**Figure 3:**
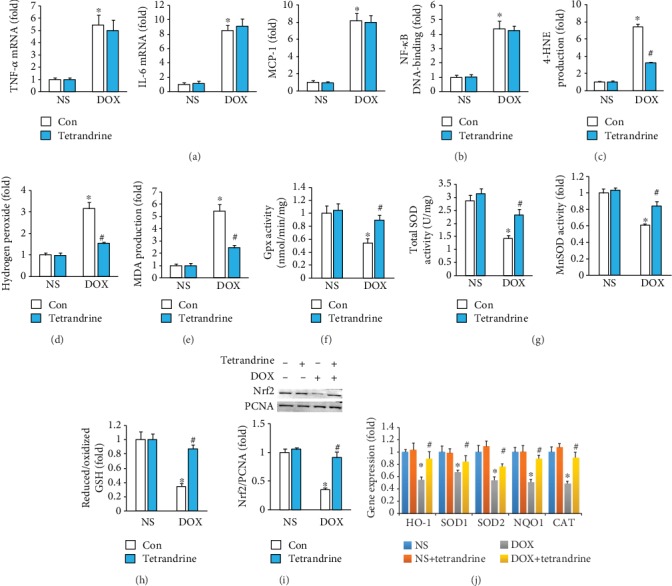
Tetrandrine treatment reduced oxidative stress in DOX-treated mice. (a) The mRNA levels of inflammatory factors in the hearts (*n* = 6). (b) NF-*κ*B activation (*n* = 6). (c–e) The levels of 4-HNE, hydrogen peroxide, and MDA in the hearts (*n* = 6). (f, g) Gpx, total SOD, and MnSOD activities in the hearts (*n* = 6). (h) Reduced/oxidized GSH (*n* = 6). (i) Nuclear Nrf2 protein expression (*n* = 6). (j) Gene expression of target genes (*n* = 6). ^∗^*P* < 0.05 compared with the saline group. ^#^*P* < 0.05 compared with mice after DOX injection.

Inflammation accumulation is a key landmark of DOX-induced cardiotoxicity [19]. Thus, we first evaluated alterations in myocardial inflammation after tetrandrine treatment. We found that the mRNA levels of tumor necrosis factor- (TNF-) *α*, interleukin- (IL-) 6, and monocyte chemotactic protein- (MCP-) 1 were significantly increased, and these increases could not be suppressed by tetrandrine treatment (Figure 3(a)). Further analysis revealed that tetrandrine treatment cannot block the activation of NF-*κ*B in response to DOX (Figure 3(b)). DOX injection significantly increased the production of 4-HNE, hydrogen peroxide, and MDA, and these increases were blocked by tetrandrine treatment (Figures 3(c)–3(e)). DOX injection resulted in the reduction of Gpx activity, total SOD activity, MnSOD activity, and reduced/oxidized GSH (Figures 3(e)–3(h)). After tetrandrine treatment, these reductions were largely blocked in DOX-injected mice (Figures 3(e)–3(h)). Western blot analysis indicated that the decreased nuclear Nrf2 protein expression in response to DOX was restored after tetrandrine treatment (Figure 3(i)). Further real-time PCR found that the downstream target of Nrf2 was markedly decreased in the DOX-treated group, and tetrandrine treatment almost restored the expression of these genes to normal levels (Figure 3(j)).

### 3.4. Tetrandrine Treatment Attenuated DOX-Induced Myocardial Apoptosis in Mice

**Figure 4:**
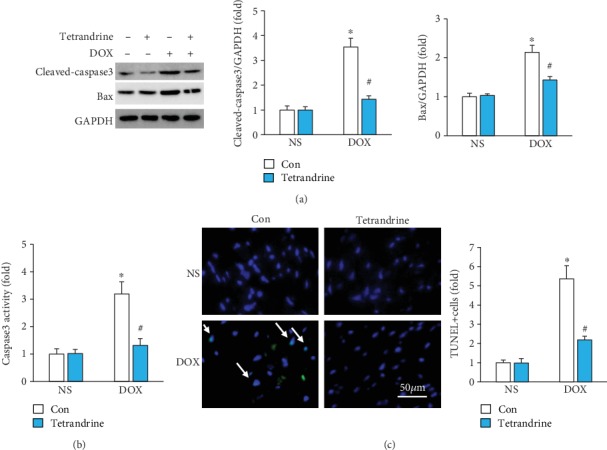
Tetrandrine treatment induced myocardial apoptosis in DOX-treated mice. (a) Protein expression of Bax and cleaved caspase3 in the hearts (*n* = 6). (b) Caspase3 activity (*n* = 6). (c) TUNEL staining (*n* = 6). ^∗^*P* < 0.05 compared with the saline group. ^#^*P* < 0.05 compared with mice after DOX injection.

The protein expression of the proapoptotic factor Bax and cleaved caspase3 were significantly increased in DOX-treated hearts; however, these changes were significantly attenuated by tetrandrine (Figure 4(a)). Subsequent analysis of caspase3 activity also revealed that tetrandrine decreased casapse3 activity in DOX-treated mice (Figure 4(b)). Next, we performed TUNEL staining to determine whether tetrandrine could reduce DOX-induced myocardial apoptosis. As shown in Figure 4(c), DOX treatment significantly increased the number of apoptotic cells in DOX-treated hearts; however, the increase in apoptotic cells was significantly less in tetrandrine-treated hearts than in hearts treated with DOX alone (Figure 4(c)).

### 3.5. Nrf2 Deficiency Antagonized the Protective Effects of Tetrandrine In Vitro

**Figure 5:**
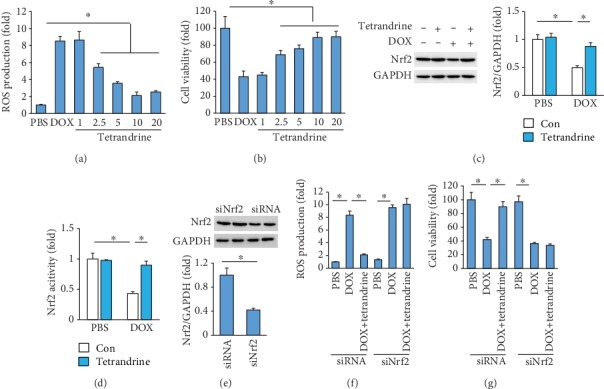
Tetrandrine provided cardioprotection by activating the Nrf2 signaling pathway. (a, b) ROS production and cell viability after DOX exposure (*n* = 6). (c, d) Nrf2 protein expression and activity (*n* = 6). (e) Nrf2 protein expression (*n* = 6). (f, g) ROS production and cell viability after DOX exposure (*n* = 6). ^∗^*P* < 0.05 vs. the matched control.

The data in our study showed that DOX markedly increased the levels of ROS, and this effect was significantly blocked by tetrandrine treatment in a dose-dependent manner (Figure 5(a)). The decreased cell viability in response to DOX was also improved by tetrandrine treatment in a dose-dependent manner (Figure 5(b)). Further analysis showed that tetrandrine treatment almost restored the expression of Nrf2 in the DOX-treated group to the normal level (Figure 5(c)). In addition, tetrandrine treatment also increased Nrf2 transcription activity after DOX exposure (Figure 5(d)). The protection provided by tetrandrine against ROS protection and cell loss was abolished by Nrf2 deficiency (Figures 5(e)–5(g)).

### 3.6. The Protective Effects of Tetrandrine Were Reversed by Nrf2 Deficiency in Mice

**Figure 6:**
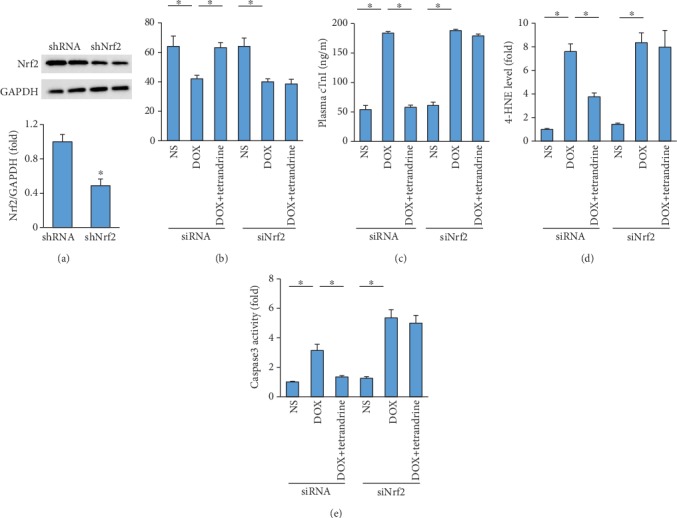
Tetrandrine did not provide cardiac protection in Nrf2-deficient mice. (a) Nrf2 protein expression (*n* = 6). (b) EF (*n* = 9). (c) The levels of cTnI (*n* = 6). (d) Myocardial 4-HNE levels (*n* = 6). (e) Caspase3 activity after Nrf2 deficiency (*n* = 6). ^∗^*P* < 0.05 vs. the matched control.

To determine whether tetrandrine exerted its protective effect via the activation of Nrf2, mice were subjected to an injection of AAV9-shNrf2 or AAV9-shRNA. Six weeks after AAV9 injection, these mice were subjected to DOX injection. As indicated in our study, tetrandrine lost its protective effect against cardiac injury in mice, as reflected by EF, plasma cTnI, 4-HNE content, and caspase3 activity (Figures 6(a)–6(e)).

## 4. Discussion

The results from our current study demonstrated that tetrandrine protected against DOX-related cardiac injury in vitro and in vivo. The protective effect of tetrandrine on the toxic effects induced by DOX was mediated by the activation of Nrf2. We also found that tetrandrine inhibited DOX-related oxidative damage and apoptotic cell death in mice. The findings support the concept that tetrandrine could be a preventive and therapeutic candidate for clinical use in DOX-related acute cardiac toxicity.

Cardiac factors are the main reason for the death of cancer survivors [20]. Thus, it is of great significance to find a drug that can limit the cardiotoxicity caused by anticancer drugs. Several scavengers of ROS have been tested for their potential to limit DOX-induced cardiotoxicity but with little success [6, 21]. The low scavenging ability and/or secondary reaction with other biomolecules might explain these failed experiments [22]. Here, we found for the first time that tetrandrine could largely reduce DOX-related acute cardiac injury and improve cardiac function in mice. The abnormal oxidative damage in response to DOX was also suppressed by tetrandrine in mice. Moreover, tetrandrine has been found to suppress tumor growth [11]. Taken together, these findings indicated that tetrandrine has the potential to be used as a drug for the treatment of DOX-related acute cardiac toxicity.

DOX exposure induced higher proinflammatory cytokine production [23]. It has been reported that tetrandrine could suppress amyloid-*β*-induced inflammatory cytokines by inhibiting the NF-*κ*B pathway [24]. Inconsistent with this previous study, we found that there was no difference between the DOX and DOX+tetrandrine groups in inflammatory factor mRNA expression and NF-*κ*B activation, implying that tetrandrine exerted cardiac protection that was not mediated by the attenuation of the inflammatory response.

It has been reported that the increased production of ROS and reduced activation of antioxidant enzymes are closely associated with the development of DOX-related cardiotoxicity [25]. Moreover, the suppression of the increased oxidative damage could improve cardiac function and survival rate in DOX-treated mice [14]. In line with these previous findings, we also found that DOX increased the levels of intracellular ROS production and lipid peroxidation products and decreased SOD and Gpx activities. After tetrandrine treatment, these abnormal alterations were largely attenuated, implying that the improvement of cardiac function was partly mediated by the attenuation of oxidative stress by tetrandrine.

In response to oxidative stress, Nrf2 translocates to the nucleus to regulate the expression of antioxidant and detoxification genes [26]. It has been reported that Nrf2 expression and activity were decreased after DOX exposure, and an Nrf2 activator could attenuate DOX-induced cardiotoxicity in mice [27], indicating that the restoration of Nrf2 was a promising strategy for the treatment of DOX-induced cardiotoxicity. Here, we also found that tetrandrine increased Nrf2 protein expression and enhanced Nrf2 activity in DOX-treated hearts or cardiomyocytes. To the best of our knowledge, this is the first report describing the activation of Nrf2 by tetrandrine. To confirm that the protection of tetrandrine was mediated by Nrf2, we used siRNA to deplete Nrf2. As expected, we found that the protection provided by tetrandrine was abolished by Nrf2 deficiency in the hearts and in cardiomyocytes, suggesting that tetrandrine protected against acute cardiac injury by activating Nrf2.

There was a direct correlation between the degree of cardiac apoptosis and the severity of DOX-induced cardiac injury [28]. ROS production in response to DOX resulted in myocardial apoptotic cell death [29]. DOX could directly upregulate caspase3 in mice [30]. The data in our study showed that tetrandrine decreased Bax and cleaved caspase3 protein expression and prevented apoptotic cell death in mice, implying that the protective effect of tetrandrine was partly attributed to the attenuation of oxidative stress by tetrandrine.

In conclusion, we found that tetrandrine protected against DOX-induced acute toxicity through the activation of Nrf2 to prevent myocardial oxidative stress and apoptosis, thus improving cardiac function. This study suggested that tetrandrine is a potential drug for the treatment of DOX-induced cardiotoxicity.

## Data Availability

The data that support the findings of this study are available from the corresponding author upon reasonable request.
